# REGULATION: Cleaner Air, Longer Life

**Published:** 2009-04

**Authors:** Carol Potera

Since the 1970s, a variety of measures for air pollution control have been introduced in an effort to improve air quality throughout the United States. Many epidemiologic studies already support the view that substantial health benefits—including a lower risk of cardiopulmonary disease and death—are derived from better air quality. A new study now suggests that improvements in U.S. air quality during the early 1980s and late 1990s increased life expectancy by several months.

First author C. Arden Pope III, a Brigham Young University economics professor, and colleagues Douglas W. Dockery and Majid Ezzati of the Harvard School of Public Health focused on particles smaller than 2.5 micrometers in diameter (PM_2.5_), which are created by fuel combustion in vehicles and power plants, among other sources. Pope says vehicle emissions tend to be the greatest local source of PM_2.5_, whereas power plants act more regionally because pollutants from smoke stacks travel farther. Numerous studies have linked PM_2.5_ with increased respiratory and cardiovascular illness and premature death.

As part of an extended analysis of the American Cancer Society prospective cohort study, the team collected air‐quality data from two 5‐year time periods for 51 U.S. metro areas spanning all geographic regions of the nation. The first data set was collected under the U.S. Environmental Protection Agency’s (EPA) Inhalable Particle Monitoring Network from 1979 through 1983. The second set, obtained through the EPA’s National Ambient Air Quality Standards for PM_2.5_, was collected from 1997 through 2001. Statistical regression models were used to evaluate changes in PM_2.5_ pollution and life expectancy, as well as to account for declines in cigarette smoking and other factors known to affect life expectancy.

As reported in the 22 January 2009 issue of the *New England Journal of Medicine* (*NEJM*), the team found that when PM_2.5_ averaged over 24 hours dropped by 10 μg/m^3^ between the two study periods, life expectancy rose by about 7 months, with a range of 6.6–12.1 months depending on the statistical model used. In those cities with the greatest drop in PM_2.5_ pollution (up to 14 μg/m^3^), life expectancy increased by almost 10 months. Overall, life expectancy increased by an average 2.7 years across the time frame studied.

“Any way you model this reasonably, you get roughly the same results,” Pope says. A 7‐month increase in life expectancy “is stunningly large when you consider no medical intervention took place,” he adds.

A variety of national and local events led to reductions in PM_2.5_ since levels were first monitored in 1980. For instance, all cities in the study benefited from emission controls on vehicles such as catalytic converters, which target pollutants that are precursors to PM_2.5_. Regulation of wood‐burning stoves and fireplaces helped to clean up the air in the Pacific Northwest. Until now, communities nationwide could only assume that air‐quality regulations would translate into increased longevity. “[This study] provides evidence that when actions are taken to reduce particulate air pollution in communities, it increases predicted life expectancy,” says environmental health professor Michael Brauer of the University of British Columbia, Vancouver.

An even larger study of the same ACS cohort, published in the 12 March 2009 issue of *NEJM* by Pope and coauthors including Michael Jerrett of the University of California, Berkeley, followed nearly 450,000 people for the same two‐decade period but this time covered 96 U.S. metro areas. After analyzing associations between the risk of death and levels of both ozone and PM_2.5_, the researchers concluded that “the risk of dying from a respiratory cause is more than three times as great in the metropolitan areas with the highest ozone concentrations as in those with the lowest ozone concentrations.” The report also reiterated the PM_2.5_ findings reported in the earlier *NEJM* article.

The studies’ findings affirm the benefits of regulatory efforts aimed at limiting exposure to both ozone and PM_2.5_. Of the first article, Dan Costa, EPA national program director for air research, says, “This study shows that PM reductions due to responses to regulatory actions are having the impact desired—protecting public health. It provides plenty of winds to our sails that we are doing the right thing, and monies spent on regulations have been worth it.”

## Figures and Tables

**Figure:**
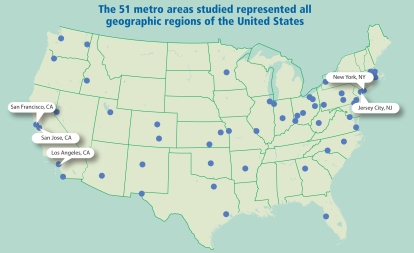
The 51 metro areas studied represented all geographic regions of the United States

**Table:** U.S. Metro Areas with the Highest Increase in Life Expectancy Associated with PM _2.5_ Reductions

Metro Area	Reduction in PM_2.5_ levels (μg/m^3^ )	Change in Life Expectancy (yr)
**New York, NY** (4 counties)	9	4.6
**Jersey City, NJ** (1 county)	4.7	4.5
**San Francisco, CA** (3 counties)	3.9	4.5
**San Jose, CA** (1 county)	3.2	4.2
**Los Angeles, CA** (1 county)	6.6	4.1

Adapted from: Pope CA III, Ezzati M, Dockery DW. Fine-particulate air pollution and life expectancy in the United States. N Engl J Med 360:376–386 (2009).

